# Novel skin phenotypes revealed by a genome-wide mouse reverse genetic screen

**DOI:** 10.1038/ncomms4540

**Published:** 2014-04-11

**Authors:** Kifayathullah Liakath-Ali, Valerie E. Vancollie, Emma Heath, Damian P. Smedley, Jeanne Estabel, David Sunter, Tia DiTommaso, Jacqueline K. White, Ramiro Ramirez-Solis, Ian Smyth, Karen P. Steel, Fiona M. Watt

**Affiliations:** 1 Centre for Stem Cells and Regenerative Medicine, King’s College London, Guy’s Hospital, London SE1 9RT, UK; 2 Department of Biochemistry, University of Cambridge, Tennis Court Road, Cambridge CB2 1QW, UK; 3 Wellcome Trust—Medical Research Council Stem Cell Institute, University of Cambridge, Tennis Court Road, Cambridge CB2 1QR, UK; 4 Wellcome Trust Sanger Institute, Genome Campus, Hinxton, Cambridge CB10 1SA, UK; 5 Department of Anatomy and Developmental Biology, Monash University, Wellington Road, Clayton, Victoria 3800, Australia; 6 Wolfson Centre for Age-Related Diseases, King’s College London, Guy's Campus, London SE1 1UL, UK; 7 Present address: Brigham Regenerative Medicine Center, Brigham and Women's Hospital, Boston, Massachusetts 02115, USA

## Abstract

Permanent stop-and-shop large-scale mouse mutant resources provide an excellent platform to decipher tissue phenogenomics. Here we analyse skin from 538 knockout mouse mutants generated by the Sanger Institute Mouse Genetics Project. We optimize immunolabelling of tail epidermal wholemounts to allow systematic annotation of hair follicle, sebaceous gland and interfollicular epidermal abnormalities using ontology terms from the Mammalian Phenotype Ontology. Of the 50 mutants with an epidermal phenotype, 9 map to human genetic conditions with skin abnormalities. Some mutant genes are expressed in the skin, whereas others are not, indicating systemic effects. One phenotype is affected by diet and several are incompletely penetrant. In-depth analysis of three mutants, *Krt76, Myo5a* (a model of human Griscelli syndrome) and *Mysm1*, provides validation of the screen. Our study is the first large-scale genome-wide tissue phenotype screen from the International Knockout Mouse Consortium and provides an open access resource for the scientific community.

## Abstract (web summary)

Large-scale efforts are put into the generation of knockout mutant mice for many individual genes. Here, the authors systematically screen skin from 538 mutant mice and identify 50 mutants with epidermal phenotypes, of which 9 are also associated with human skin defects.

## Graphical abstract

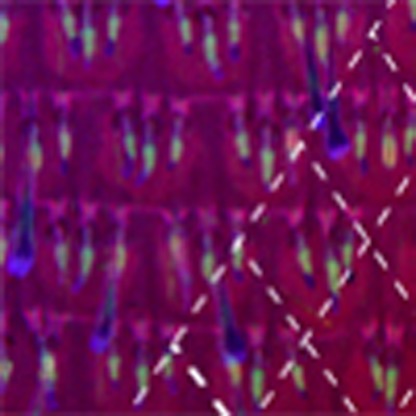

The large amount of genetic information accumulated in the post-genomic era needs to be transformed into knowledge^1^. To study the functions of large numbers of genes *in vivo*^2^ requires a shift from gene-specific to genome-wide approaches. Previous studies have catalogued libraries of mutant mice that lack specific classes of proteins^3^ or exhibit behavioural phenotypes^4^. However, a large-scale screen (>100 mutants) for phenotypes in a specific tissue via targeted gene knockouts has not been reported previously.

For many years, researchers have been studying genes involved in epidermal homeostasis with an emphasis on stem cells and disease^5^,^6^. Since the first description of the hairless mouse in 1924, many spontaneous and random mutations with skin phenotypes have been studied by classical (forward) genetic approaches^7^. Owing to technical limitations, a large-scale high-throughput systematic functional screen for genes involved in skin homeostasis was not feasible until recently. Such a screen would facilitate the identification of novel genes that are involved in skin homeostasis, cancer, aging, infection, wound repair and sensation.

Genome-wide approaches to epidermal function include short interfering RNA-based genetic screens in cultured human epidermal cells^8^ and RNA interference-mediated gene knockdown via *in utero* microinjection of lentiviral vectors^9^. Neither approach can identify effects of genes that are not expressed in the epidermis but influence epidermal homeostasis by local or systemic signalling. A further challenge is that it is extremely laborious to assess phenotypes in large areas of adult skin by relying on conventional histological sections. This can be overcome by preparing wholemounts of intact tail epidermis^10^ in which the interfollicular epidermis (IFE), sebaceous glands (SGs) and hair follicles (HFs) are easily discerned. Virtually all epidermal stem cell populations express keratin (K)14 and one well-defined stem cell reservoir is in a region known as the HF bulge, which expresses K15 and is readily identified by its location^11^.

We have employed a high-throughput reverse genetics screen to identify skin phenotypes in mutant mice generated from the Sanger Institute Mouse Genetics Project (MGP)^12^,^13^. Identifying the phenotype correlations of genes involved in skin homeostasis paves the way to interrogate functional genetic interactions and find therapeutic targets for human skin diseases.

## Results

### Screen strategy

The screening strategy was designed to analyse the tail epidermis of mutants available from the terminal phenotyping pipeline of the MGP. The screen was typically performed on viable adult homozygote mutants, or heterozygotes in the case of mutants with homozygote embryonic lethality. We analysed the skin without prior knowledge of the genotype. In principle, the screen could have been carried out with a single skin sample per genotype. However, we typically received two samples of each genotype, although we did not necessarily receive them at the same time. Pipeline design, generation of mice and primary phenotyping are described elsewhere^12^,^13^.

**Figure 1:**
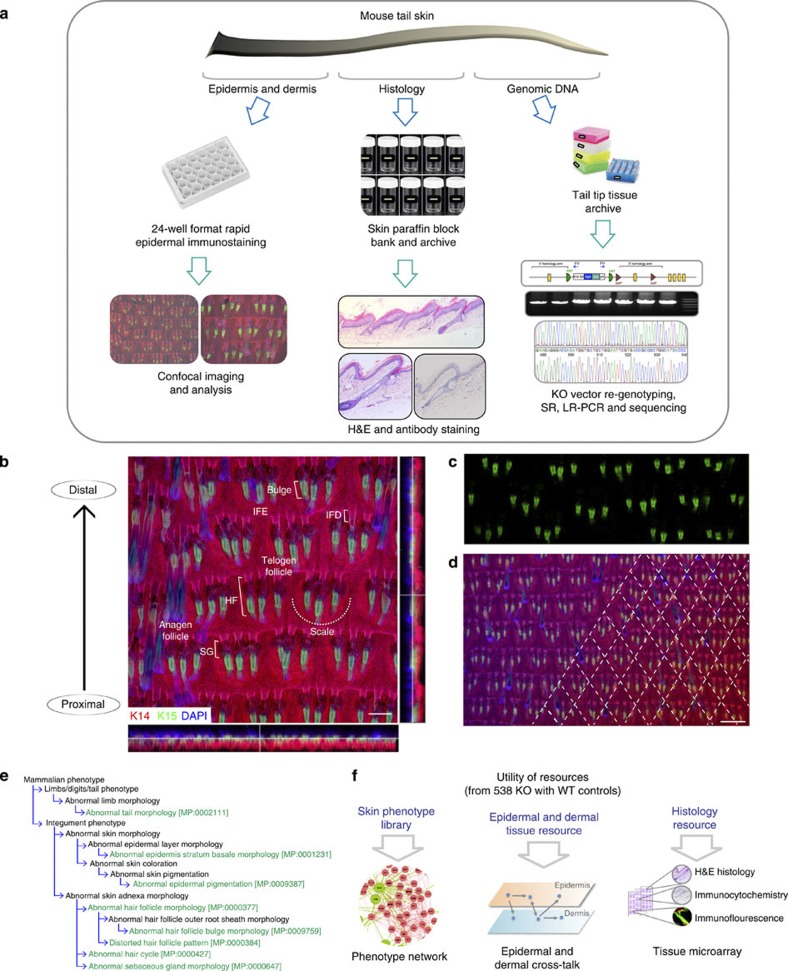
Skin screen strategy and exploitation of the resource. (**a**) Screen strategy. On receipt, tail skin was divided into three parts: one was processed for epidermal wholemount immunostaining, one for paraffin conventional histology and one for genomic DNA extraction. (**b**) WT epidermal wholemount image labelled for K14 (red), K15 (green) and DAPI (blue) showing hair follicle (HF), interfollicular epidermis (IFE), infundibulum (IFD), sebaceous gland (SG), bulge, scale and anagen and telogen HFs. (**c**) Single channel (K15-488) image showing labelling of HF bulge. (**d**) Low magnification image of epidermis emphasizing patterned arrangement of HF triplets (one triplet per diamond marked with dashed lines). (**e**) MP ontology tree. MP terms in green were used to annotate skin phenotypes in this study. (**f**) Schema shows how resources generated from this study can be further utilized. Scale bars, 100 μm (**b**,**c**); 200 μm (**d**).

To facilitate analysis of large numbers of samples, the tail epidermal wholemount protocol was modified to reduce the time required for labelling and increase the number of samples that could be labelled simultaneously (Fig. 1a). Tissue was simultaneously labelled with fluorescently conjugated antibodies to K14 and K15 and subsequently counterstained with 4',6-diamidino-2-phenylindole (DAPI) nuclear counterstain. Epidermal sheets were labelled in individual wells of 24-well plates and up to 3 plates (72 specimens) were labelled simultaneously. In addition, pieces of intact tail skin were paraffin embedded for detailed histology and DNA was extracted for verification of genotype if required (Fig. 1a).

Wholemounts were analysed by non-automated high-content confocal imaging (Fig. 1b–d; Supplementary Movie 1). In addition, any obvious macroscopic abnormalities, such as altered skin pigmentation or abnormal tail length, were recorded. For wild-type (WT) reference data, a panel of tail epidermal wholemount images from multiple WT genetic strains was generated for comparison with the respective mutant lines (Supplementary Fig. 1; see Methods). Many previous reports have highlighted the need to use specific vocabularies for consistent phenotypic annotation of mammals^14^,^15^. We therefore used existing mammalian phenotype (MP) terms to classify our phenotypes under the broad ‘integument phenotype’ and ‘limbs/digits/tail phenotype’ categories of the Mouse Genome Informatics database ( http://www.informatics.jax.org/searches/MP_form.shtml; Fig. 1e).

Eight specific MP terms were used to annotate epidermal abnormalities (Fig. 1e). ‘Abnormal tail morphology’ was based on macroscopic evaluation (for example, thickened, kinked, scaly) but often manifested in abnormalities that were also detectable in epidermal wholemounts. In the tail, HFs are typically arranged in groups of three (triplets) and in staggered rows; when this pattern was abnormal, the phenotype was scored as ‘distorted HF pattern’. Since the hair growth cycle is not synchronized in mice of the ages analysed (older than 13 weeks), the cycle was scored as ‘abnormal hair cycle’ if the majority of hair HFs were in anagen or telogen. When bulge size or K15 expression was altered, the skin was scored as ‘abnormal HF bulge morphology’ and when the shapes of individual follicles were distorted, we scored the phenotype as ‘abnormal HF morphology’. We also scored abnormalities in the tail IFE, including scale versus interscale differentiation^16^ (‘abnormal epidermis stratum basale morphology’), and in SG morphology (abnormal size; irregular surface). In tail epidermis, melanocytes, identified by their dark colour, are normally confined to the HF or scale IFE and when this pattern was disrupted, the phenotype was scored as ‘abnormal epidermal pigmentation’. The results were entered into a structured tick sheet before annotation and deposition at the Sanger Mouse Resources Portal ( http://www.sanger.ac.uk/mouseportal/). This open access resource can be exploited to interrogate many aspects in skin biology, including information on the macroscopic appearance of living mice and histology of the skin from sites other than tail (Fig. 1f).

### Novel skin phenotypes

Mutant mouse lines for a total of 557 alleles belonging to 538 unselected genes covering all mouse chromosomes except Y were analysed (Supplementary Data 1 and 2, and Supplementary Fig. 2a) along with matched WT control samples. The specific allele types are listed in Supplementary Data 2 and homozygotes are designated ‘*−/−*’ in the text for simplicity. Gene ontology (GO) predicted that the majority of the encoded proteins are located in the cytoplasm and nucleus, with many involved in chromosome and chromatin organization. Large numbers of proteins were significantly enriched for GO terms corresponding to catalytic (28%; log_10_ *P* value, −13.7356) and protein binding (30%; log_10_ *P* value, −19.6965) functions (Supplementary Fig. 2b and Supplementary Data 2).

**Figure 2:**
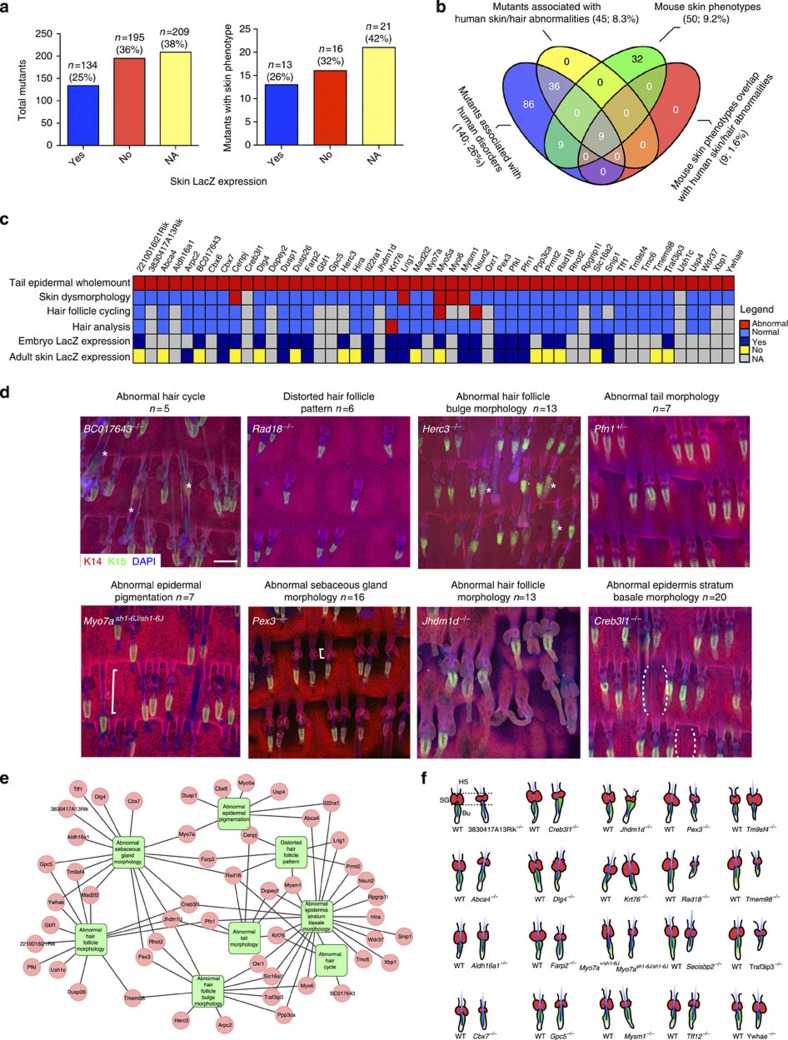
Phenotype summary. (**a**) *lacZ* reporter expression in skin. NA, not applicable because not determined. Left hand panel shows data for all mutants. Right hand panel shows data for those mutants with a skin phenotype. (**b**) Venn diagram showing overlap between the 50 genes identified with skin phenotypes in the screen, genes in the screen that are associated with any human disease or human skin/hair abnormalities, and genes associated with both mouse and human skin/hair abnormalities. (**c**) Heatmap showing all 50 phenotypic hits in the tail epidermal wholemount screen (top row), LacZ expression data from the MGP (bottom two rows) and the results of three macroscopic skin-related tests available through the MGP (middle rows). (**d**) Representative tail epidermal wholemount images of phenotypic hits from eight categories annotated using MP terms. Images are 3D maximum projections, except for *Pex3*^*−/−*^, which shows a single stack. (**e**) Association of each hit with the eight phenotypic categories annotated using MP terms. (**f**) Outlines of individual mutant HF and SG (red) compared with WT. WT and mutants are matched for genetic background. Scale bars, 100 μm.

Most mice were engineered to express a *lacZ* reporter via the promoter of the deleted gene^12^. Skin *lacZ* expression was observed in 25% of mutant lines (*n*=134) and was absent in 36% (*n*=195). In 38% (*n*=209) *lacZ* expression was either not evaluated or not determined, because the mice were deemed unsuitable for analysis or else a read-out of *lacZ* expression could not be obtained (Fig. 2a). We observed abnormalities in 9.2% (*n*=50) of mutant lines. Skin *lacZ* expression was observed in 26% (13/50) and absent in 32% (16/50) of mutants with epidermal phenotypes (Fig. 2a, Supplementary Data 3 and 4, and Supplementary Fig. 3).

Fourteen percent of hits (*n*=7) from the tail epidermal wholemount screen were also found independently in the MGP screen of live mice for macroscopically observable skin-related parameters (skin dysmorphology, HF cycling and hair analysis; Fig. 2c; data available from http://www.sanger.ac.uk/mouseportal). Two of the hits in our screen, leucine-rich transmembrane protein *Lrig1* (ref. 17) and the RNA methyltransferase *Nsun2* (ref. 18), have previously been identified as playing a role in epidermal homeostasis and thus served as positive controls (Fig. 2c and Supplementary Fig. 4). Further information about skin phenotypes is available via the portal under the heading ‘skin histopathology’ or by searching for individual genes under the category ‘integument’.

Representative images of each category of tail epidermal abnormality are shown in Fig. 2d. Several mutants exhibited abnormalities in more than one MP term. For example, the *Jhdm1d*^*−/−*^ (also known as Kdm7a) phenotype was recorded as ‘abnormal HF morphology’ and also ‘abnormal tail morphology’, ‘abnormal SG morphology’ and ‘abnormal HF bulge morphology’ (Fig. 2d,e). Figure 2f shows outlines of individual HF with associated SG in the subset of mutants that showed HF/SG phenotypes, together with WT examples for comparison.

‘Abnormal epidermal stratum basale morphology’ and ‘abnormal SG morphology’ (40%; *n*=20 and 34%; *n*=17, respectively) represented the maximum number of ‘hits’. The IFE abnormalities included loss of distinct scale/interscale boundaries and crowding of basal layer cells, indicative of increased proliferation. In some cases, such as *Creb3l1* (Fig. 2d), both abnormalities were present; since the scales form through more rapid basal cell proliferation than in interscales^16^, lack of scales could potentially be a consequence of IFE hyperproliferation. SGs are sac-like structures emerging from the junctional zone of HF with two to three lobes^19^. Most of the abnormalities related to SG size (for example, *Pex3*^*−/−*^ had small SG and *Krt76*^*−/−*^ had large SG), but *Dlg4*^*−/−*^ and *Myo7a*^*−/−*^ mutants displayed SG with irregular surfaces (Fig. 2f).

One of the most striking features of the HF phenotypes was that they were not obligatorily linked to one another. Thus, a mutant with abnormal bulge morphology (for example, *Herc3*^*−/−*^) could have a normal hair cycle. Conversely, the hair cycle could be perturbed in an otherwise normal epidermis (for example, *BC017643*^*−/−*^; Fig. 2d,e). In contrast, abnormal tail morphology was always linked to other epidermal phenotypes (Fig. 2e).

The mutant mice were generated as part of two different analysis pipelines, one of which included a high-fat diet while the other involved a standard chow diet. In most cases, it was not possible to look for an influence of diet on mutant epidermis, although we saw no effect of diet on WT control tail epidermis (see Methods). However, *Tm9sf4*^*−/−*^ mutants went through both pipelines and an effect of diet was observed. Mutant mice fed on chow had smaller SGs than WT, whereas mutants fed on the high-fat diet did not (Supplementary Fig. 5).

We also observed incomplete penetrance of phenotype in some mutants, in particular *Rad18* and *Jhdm1d* (Fig. 2d and Supplementary Fig. 6), both of which exhibited strong phenotypes in four of eight categories examined but only for one mouse per genotype, the others being phenotypically normal (Supplementary Data 4). This is of interest because some mutations associated with skin abnormalities have incomplete penetrance among human populations^20^.

Most mice analysed were female, since they are less likely to fight and sustain skin wounds that could confound the analysis. To control for potential sexual dimorphism in skin structures, we analysed genes involved in oestrogen signalling. We extracted a list of 368 genes from the oestrogen responsive genes database^21^ ( http://datam.i2r.a-star.edu.sg/ergdbV2/). We found that 12 of the 538 genes in our screen are oestrogen responsive; however, none scored positive for any epidermal abnormalities (Supplementary Data 1).

### Association with human disorders and signalling pathways

We used the PhenExplorer application^22^ ( http://www.compbio.charite.de/phenexplorer) to examine whether any of the mutants in our screen were already linked to human diseases, in particular those with a skin phenotype. The key advantage of the PhenExplorer application is that users can search individual phenotypic features associated with any human disease by visualizing specific phenotypic terms with unique IDs. As an alternative strategy, to ensure we had not missed any known rare diseases or skin phenotype associations, we also interrogated the Human Phenotype Ontologies database, diseases of Online Mendelian Inheritance in Man and Orphanet (rare diseases and orphan drugs) databases. Twenty-six percent (*n*=140) of the mouse-mutant lines we analysed (*n*=538) were linked with human disorders, including 18 out of 50 mutants with an epidermal phenotype. Of those 18 mutants, 9 were associated with a human skin phenotype. These mutants provide useful experimental models to study the pathophysiology of human skin diseases or test potential therapeutics (Fig. 2b and Supplementary Data 3).

**Figure 3:**
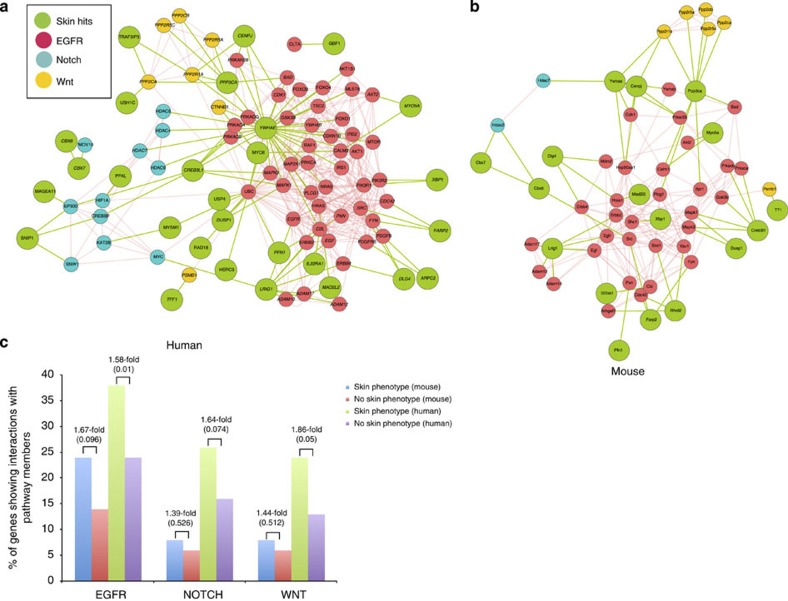
Potential interactions of mutant genes with the Egfr, Notch and Wnt pathways. (**a**) Human, (**b**) mouse. Genes common in human and mouse are italicized. (**c**) Number of mutants interacting with human and mouse Egfr, Notch and Wnt pathways, showing statistical analysis of enrichment by an exact binomial probability test.

Egfr, Wnt and Notch signalling pathways regulate many aspects of epidermal homeostasis in humans and mice^23^. We determined whether the 50 genes with an epidermal phenotype were enriched for interactions with these pathways relative to the background population of 488 genes that did not (Fig. 3a–c; Supplementary Fig. 7). A binomial probability calculation using the background rate of interactions of the 487 genes was performed to test whether the observed enrichments for the 50 skin genes were statistically significant. There was statistically significant enrichment for EGFR (epidermal growth factor receptor; *P*-value<0.05, by exact binomial probability) interactions, and borderline enrichment (*P* values from 0.05 to 0.1, by exact binomial probability) for the NOTCH and WNT pathways in humans, where more interaction data are available compared with mice. The main potential links to the WNT pathway were via a family of protein phosphatases that are of unknown function in skin, while the potential NOTCH interactors included histone deacetylases. In the case of EGFR interactions, we found associations with many genes known to affect epidermal differentiation and homeostasis, including cell cycle regulators, Foxo transcription factors and the MAPK signalling pathway^24^.

We also performed an unbiased search for cellular processes and phenotypes affected by the mutant genes. Enrichment analysis was performed using the ToppFun tool in the ToppGene suite ( http://www.toppgene.cchmc.org) on all 50 skin phenotype hits annotated according to the specific MP terms (Supplementary Table 1). The default Bonferroni method for multiple testing was used to output results with a false discovery rate below 0.05. The background data sets used to calculate enrichment were all those supplied by ToppGene: for example, protein functions represented by all human gene GO annotations, disease–gene associations from Online Mendelian Inheritance in Man and phenotype annotations from the Human Phenotype Ontologies and Mouse Genome Informatics databases. The genes linked to abnormal skin pigmentation had known functions in pigmentation, such as granule transport (6.104E−3), and were associated with abnormal eye pigmentation (2.460E−2). The other seven MP terms yielded only vestibular hair cell degeneration (4.214E−2) as a previously identified phenotype.

As further validation of the screen, we explored the phenotype of three hits in greater depth. *Krt76* was selected because it is a keratin and so any effects should be intrinsic to epidermal cells^25^. The type V myosin *Myo5a* was examined because it is mutated in a human condition called Griscelli syndrome that has skin phenotypes^26^. The histone H2A deubiquitinase *Mysm1* was selected because although it had a striking skin phenotype its reported functions are in the haemopoietic system^27^,^28^.

### *Krt76* mutants have aberrant epidermis and SG

Keratin intermediate filament proteins protect epithelial cells from mechanical and non-mechanical stresses^29^. *Krt76* is a type II intermediate filament ( http://www.interfil.org) that is downregulated in human oral carcinoma^25^. *Krt76*^*−/−*^ mice exhibit preneoplastic changes in the gingivobuccal epithelium^25^, but *Krt76* function in the epidermis is unknown.

**Figure 4:**
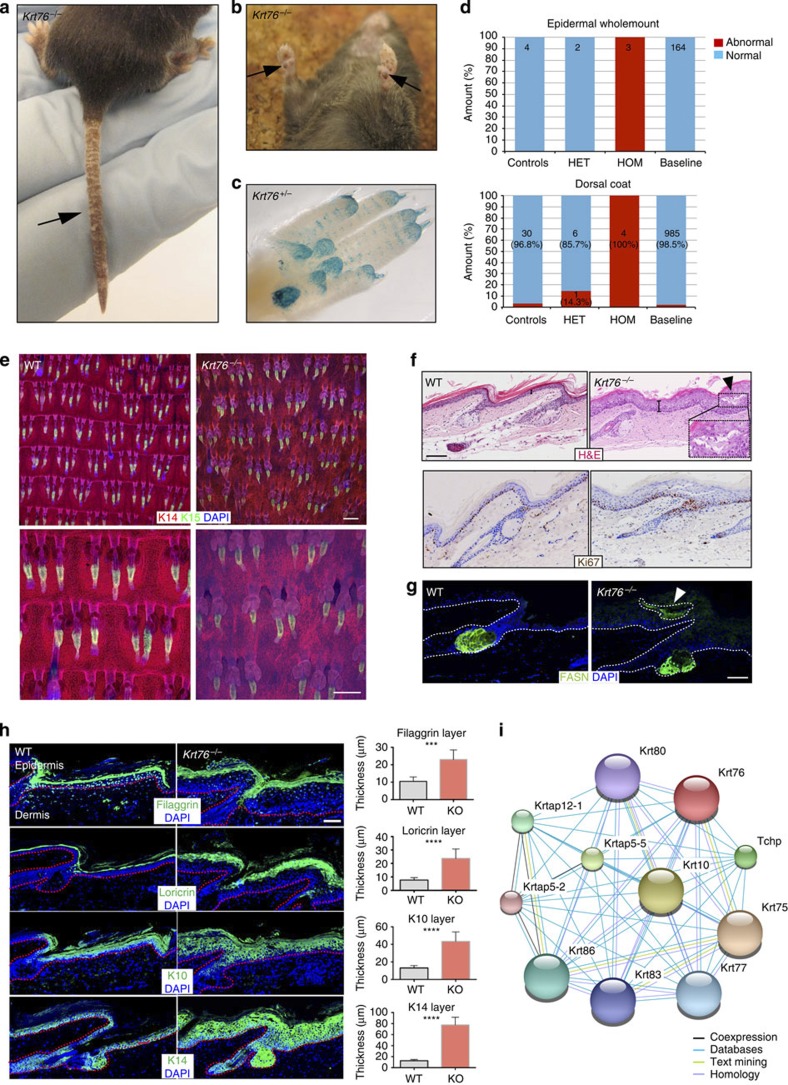
*Krt76*^*−/−*^ phenotype. (**a**) Flaky tail skin (arrow). (**b**) Darkly pigmented forepaws (arrows). (**c**) *lacZ* reporter expression in footpad (downloaded from Sanger Mouse Portal). (**d**) Stack bars showing proportion of *Krt76*^*−/−*^ mice with abnormalities observed in epidermal wholemounts or dorsal coat colour. HET, heterozygous; HOM, homozygous; control, WT mice run each week through the Sanger MGP to ensure that all testing conditions are normal; baseline, all controls were run for a particular background, pipeline and diet combination. (**e**) Confocal epidermal wholemount images of WT and *Krt76*^*−/−*^ mice. (**f**) Histological sections stained with haematoxylin and eosin (upper panel) and Ki67 (lower panel). Ectopic sebocytes in the IFE (arrowhead) are shown at higher magnification in the insert. Brackets indicate IFE thickness. (**g**) Immunostaining for the SG differentiation marker, FASN. Ectopic sebocytes are marked by arrowheads. (**h**) Immunostaining for the markers indicated. Thickness of each layer was quantified using Fiji (Image J) software. Stack bars show results of unpaired *t*-test. Error bars denote mean (*n*=3) with s.d., ***P<0.0002, ****P<0.0001. (**i**) STRING network image shows possible interaction partners of Krt76. Scale bars, 50 μm (upper panel) and 100 μm (lower panel) (**e**); 200 μm (**f**,**g**); 100 μm (**h**).

*Krt76*^*−/−*^ mice had flaky tails and both the tail and footpads were darkly pigmented (Fig. 4a,b). *lacZ* reporter expression was observed in footpads (Fig. 4c). *Krt76* heterozygotes had no obvious skin abnormalities, although an abnormal dorsal hair coat was observed in a small percentage (Fig. 4d). Tail epidermal wholemounts were abnormal in four phenotypic categories, including abnormal stratum basale and SG morphology (Fig. 4e). Analysis of haematoxylin and eosin-stained tail skin revealed epidermal thickening and basal layer hyperproliferation (Fig. 4f). The scales were no longer clearly defined and instead the granular layer (which is normally lacking in the scale) was continuous. The tail IFE also contained clusters of large cells with pale pink cytoplasm (Fig. 4f), resembling the ectopic sebocytes that form upon abnormal epidermal *Myc* activation^10^,^30^. These cells were confirmed as sebocytes on the basis of FASN and *SCD1* expression (Fig. 4g). Consistent with IFE hyperproliferation, there was suprabasal expression of *Krt14* and an increase in the number of suprabasal layers expressing the differentiation markers *Loricrin*, *Filaggrin* and *Krt10* (Fig. 4h). As predicted from the changes to the granular layer, *Filaggrin* labelling was continuous rather than confined to the interscale^16^.

We performed known and predicted protein–protein interaction analysis using the STRING database ( http://www.string-db.org/)^31^ to look for functional protein associations with *Krt76*. There are no experimentally recorded interactions of *Krt76,* but predicted interactions retrieved other keratins or intermediate filament-associated proteins such as *Tchp, Krt77, Krt86* and *Krt10*. Numerous genetic skin disorders are associated with keratin mutations, including *Krt10,* which is mutated in recessive epidermolytic ichthyosis with hyperkeratosis^32^ (Fig. 4i).

### *Myo5a* null mice phenocopy Griscelli syndrome

**Figure 5:**
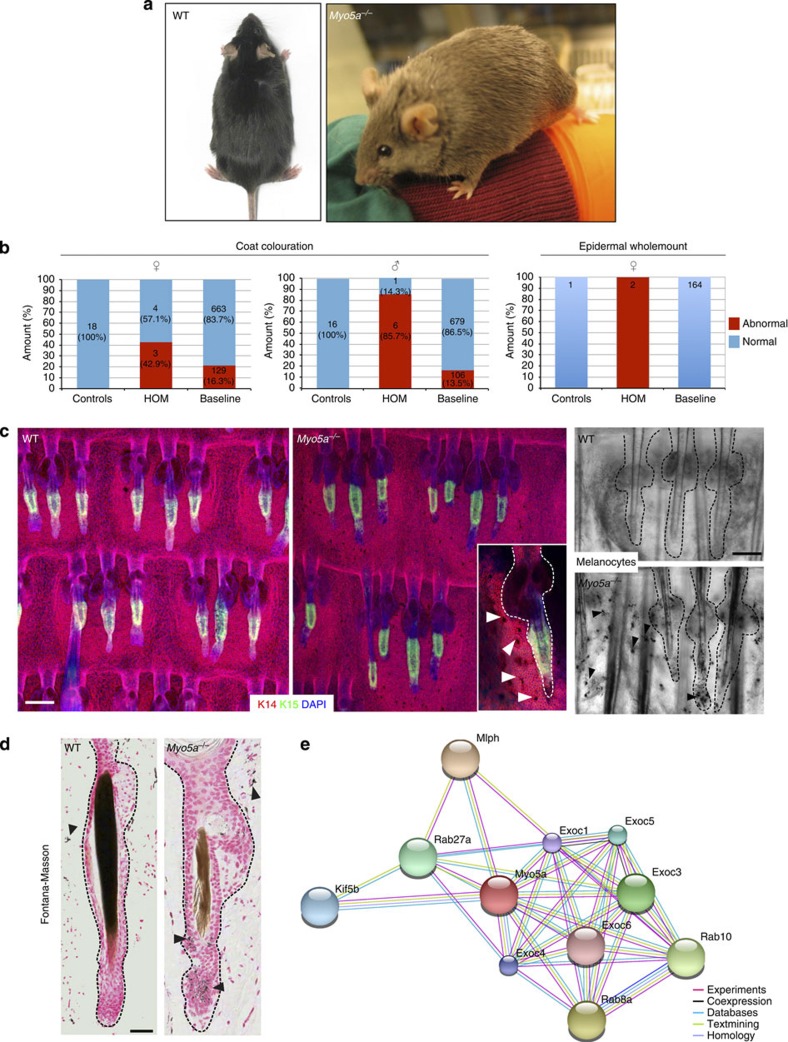
*Myo5a*^*−/−*^ phenotype. (**a**) Coat colour of WT and *Myo5a*^*−/−*^ mice on a BL6 genetic background. (**b**) Stack bar graphs shows coat colour and epidermal abnormalities. See the legend of Fig. 4d. (**c**) Epidermal wholemounts. Note abnormal melanocyte morphology in IFE interscale (arrowheads in magnified insert). Bright-field image showing melanosome clumping in *Myo5a*^*−/−*^ epidermal melanocytes. (**d**) Melanocyte-specific Fontana–Masson staining of skin sections showing aggregation of melanosomes in melanocytes in *Myo5a*^*−/−*^ HF. Note also differences in colour of WT and mutant hair shafts. Individual HF with SGs are demarcated with dashed lines. (**e**) STRING network image shows known (experimental) and predicted interaction partners of *Myo5a*. Scale bars, 100 μm (**c**); 200 μm (**d**).

Mutations affecting the actin-based motor protein MYO5A underlie a rare human genetic condition known as Griscelli syndrome^26^, which is characterized by hypopigmentation of the skin and hair. Several spontaneous mutations in the dilute locus were previously reported in mice with a lighter coat colour in a non-agouti background, leading researchers to map the *Myo5a* gene to this locus^33^. In *Myo5a*^*−/−*^ mice, the coat colour abnormality was obvious (Fig. 5a,b). Tail epidermal wholemounts (both immunostained and unstained) revealed abnormal morphology of melanocytes and increased intracellular accumulation of melanin pigment (Fig. 5c,d), consistent with previous reports that the coat-colouration phenotype in dilute mice results from abnormal melanosome transport from melanocytes into epidermal keratinocytes^34^. Abnormal pigmentation was confirmed by conventional histology (Fig. 5d). STRING network analysis revealed experimentally validated interaction partners of *Myo5a*, of which *Rab27a* and *Mlph* are known to have a role in melanosome transport in melanocytes^35^ (Fig. 5e).

We also identified abnormal epidermal pigmentation in mice with a spontaneous mutation in *Myo7a*, which had unusually dark and large melanocytes in the IFE (Fig. 2d and http://www.sanger.ac.uk/mouseportal/). Surprisingly, these mutants did not show any coat-colour abnormalities, which may reflect different genetic backgrounds of the *Myo7a* and *Myo5a* mutants (Supplementary Data 2). *Myo7a* also belongs to the actin-based family of motor proteins and is involved in melanosome transportation by binding to *Rab27a* in retinal pigment epithelia, the same mechanism by which *Myo5a* contributes to epidermal pigmentation^34^,^36^,^37^.

### Deletion of *Mysm1* leads to abnormal HF patterning

**Figure 6:**
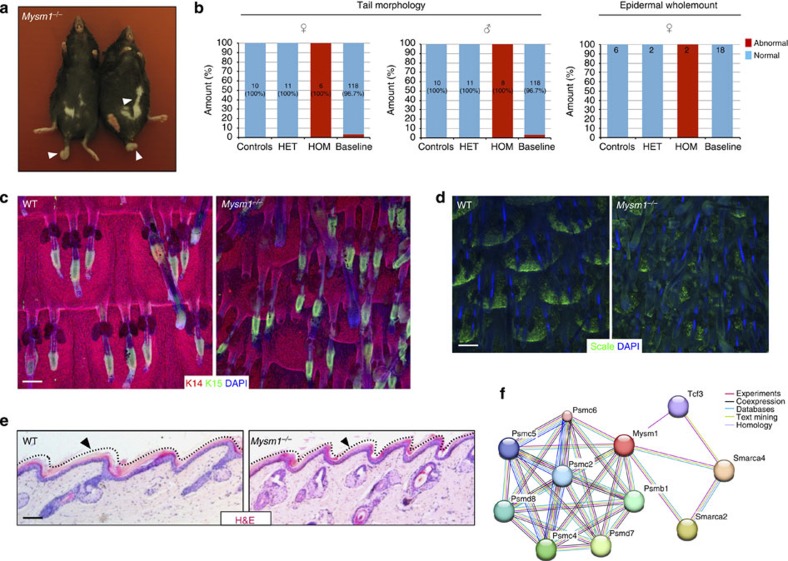
*Mysm1*^*−/−*^ phenotype. (**a**) Adult *Mysm1*^*−/−*^ homozygous mutant mice with short kinked white tails and white belly patches (arrows). (**b**) Stacked bars show incidence of abnormal tail morphology and epidermal wholemounts. See also the legend of Fig. 4d. (**c**,**d**) Epidermal wholemount images show disrupted HF pattern in *Mysm1*^*−/−*^ mice when compared with WT. In **d** wholemounts were stained with the scale-specific marker ab1653 (ref. 16). (**e**) Haematoxylin and eosin (H&E)-stained sections showing parakeratotic interscale in WT and mutant tail epidermis. (**f**) STRING network image shows known (experimental) and predicted interaction partners of *Mysm1.* Scale bars, 100 μm (**c**–**e**).

*Mysm1* is a histone H2A deubiquitinase^38^, previously identified as a positive regulator of the androgen receptor^39^. *Mysm1*^*−/−*^ mutants all had white kinky tails (Fig. 6a), and displayed abnormalities in the IFE and HF (Fig. 6b). Abnormal HF patterning was observed, with HF no longer arranged as staggered rows of triplets. In addition, the HF cycle was disturbed, with most HFs being in telogen in the specimens examined (Fig. 6c). Within the IFE, the scale pattern was completely disrupted^16^, although, in contrast to the *Krt76*^*−/−*^ mutant, regions of scale differentiation lacking the granular layer could still be observed (Fig. 6d,e). STRING network analysis identified the transcription factor *Tcf3* as an experimentally validated interactor, which is of interest, given the known role of *Tcf3* in epidermal homeostasis, where it functions in both a Wnt-dependent and Wnt-independent manner^40^ (Fig. 6f). In addition, Wnt signalling is known to regulate scale pattern^16^.

*Mysm1* mutants also had a white ‘belly spot’, which is a hallmark of melanoblast migration defects in BL6 background mice ( http://www.sanger.ac.uk/mouseportal/phenotyping/MAHN/mp-report/integument). Many similar belly spot phenotypes have been reported, with different genes involved^41^,^42^. Mysm1 is downregulated in mice with mutations influencing eye pigmentation^43^. Although the ubiquitin proteasome system is known to play a role in regulating skin pigmentation^44^, we did not observe a strong pigmentation defect in tail epidermal wholemounts.

## Discussion

Now that large numbers of mouse mutants are available through the International Knockout Mouse Consortium^45^, there is a need to screen phenotypes via correspondingly high-throughput approaches. By making phenotype data available immediately via an open access resource, results can be rapidly and widely disseminated, and potential interactions between genes or between phenotypes can be revealed. We have used this approach to carry out the first large-scale tissue-specific screen of mutants from the MGP.

Seventy-eight percent of the 250 adult mouse mutants analysed in the initial MGP screen had a phenotype affecting at least one tissue or physiological process^13^. From our screen of 538 mutants, we identified 50 genes, mutations in which led to some form of tail epidermal phenotype. As part of the screen, we collected tail skin for paraffin embedding. This has created a valuable archive from which we can create tissue microarrays, to retrospectively explore other aspects of the skin, such as innervation, connective tissue and vasculature^46^. We examined the tail skin phenotypes of three mutants in depth, with larger numbers of mice (Supplementary Data 4), and in each case validated the conclusions from the initial screen.

For most mutants a functional role in skin was not anticipated. Of those genes with an epidermal phenotype and linked *lacZ* data, approximately equal numbers were expressed or not expressed in the skin, the latter indicating a non-cell autonomous effect. The majority of mutant genes that were not expressed in skin showed phenotypes in other body systems, particularly affecting the immune system and homeostasis/metabolism ( http://www.sanger.ac.uk/mouseportal/). For example, *Mysm1* has so far been primarily implicated in regulating immune cell function, which can indirectly affect the HFs and IFE^5^. There is a precedent for this in other tissues: *Trim45* is not detected in the cartilage or bone, yet on deletion results in brittle bones and a high bone mineral content^47^.

Some mutants had multiple skin phenotypes, while others were distinct. Many mice with defects in the hair growth cycle or HF morphology have been described previously^5^. However, we found a surprisingly high incidence of SG abnormalities. For example, *Pex3* plays a role in peroxisome biogenesis^48^ and *Pex3*^*−/−*^ mice had abnormally small SGs. In humans, peroxisome deficiency results in inflammatory hair disorders, such as primary cicatricial or scarring alopecia and lichen planopilaris, with SG atrophy as a key pathological feature. It is reported that *Pex3* and its regulator peroxisome proliferator-activated receptor-γ are significantly downregulated in lichen planopilaris^49^. Another gene with a role in lipid metabolism was *Aldh16a1*, which belongs to the aldehyde dehydrogenase (ALDH) protein family. These mutants exhibited smaller SGs than WT. Previous reports suggest a role for other ALDHs in SG^50^,^51^, indicating that *Aldh16a1* may have a similar function.

Although we did not initially design the tail epidermal screen to identify pigmentation defects, these were readily observed, both macroscopically and in wholemounts. While *Myo5a* phenocopied human Griscelli syndrome, a role for *Myo7a* in epidermal pigmentation was not predicted. These two mouse models could potentially be used to investigate melanosome transfer into epidermal cells and the consequences of transfer deficiency for ultraviolet carcinogenesis. A further category of genes that are particularly interesting are those characterized by IFE hyperproliferation, since these were often associated with loss of scale patterning, supporting the concept that scale and interscale turn over at different rates and providing a potential tool for identifying the distinct pools of scale and interscale stem cells^16^. Six of nine mutants (*Abca4, Hira, Nsun2, Rpgripl1, Slc16a* and *Tmc6*) in the ‘abnormale stratum basale’ category have skin phenotypes in humans (Supplementary Data 3).

Since the mutants were unselected and covered a wide variety of biological functions, we asked whether mutants with a skin phenotype mapped to the Wnt, Notch and Egfr pathways, all of which play key roles in epidermal differentiation and homeostasis^52^,^53^. We identified four mutants, *Cenpj, Farp, Lrig1* and *Rad18,* with distorted HF patterning that are direct targets of the Egfr pathway. Two other mutants, *Mysm1* and *Prmt2*, mapped to the Wnt and NF-κB pathways, respectively.

Our study represents the first large-scale tissue-specific screen of mutant mice. The largest number of mutants screened previously was 100, in the case of bone abnormalities^47^. We believe that the data and resources generated will accelerate research in many labs with an interest in skin function and pathology. In the past, generating knockout mice was time consuming and expensive, and frequently led to duplication of effort in different labs. Our advances include high-throughput analysis of the epidermis by combining an optimized wholemount labelling protocol with phenotyping based on standard ontologies. The bottleneck in our screen has been in evaluating individual wholemounts by confocal microscopy and collecting images of mutant phenotypes by three-dimensional (3D) Z-stacks. This approach is slow, requires a considerable amount of data storage and to avoid subjective calls requires a second person to examine a subset of wholemounts.

Future epidermal wholemount screens could be improved by replacing visual inspection with automated, high-throughput, high content imaging, which would also allow us to examine more mice per genotype. When we designed the screen, in 2006, many epidermal stem cell populations had not been identified^11^. Nevertheless, the combination of K14 and K15 labelling has provided a satisfactory and robust means of identifying the major regions of the epidermis. Given the large number of mutants with abnormal SG, the next iteration of the screen could include a lipophilic dye, such as LipidTox, to enhance visualization of the SG without appreciably lengthening the staining procedure. Finally, while the MGP allows integration of multiple data sets for a given mouse mutant, the skin field would undoubtedly benefit from a platform that would facilitate integration of the skin screen with screens of *in vitro* and *in utero* epidermal knockdowns^8^,^9^, together with gene expression and genome-wide chromatin immunoprecipitation data sets. This holds great promise for skin research.

## Methods

### Mouse mutants

Mutant mice were generated by high-throughput gene targeting in embryonic stem cells^12^ by the Wellcome Trust Sanger Institute’s MGP as part of the International Knockout Mouse Consortium. Mice were maintained at the Sanger Institute, the Wellcome Trust-MRC Cambridge Stem Cell Institute or the Cancer Research UK Cambridge Research Institute with local ethical approval from each institution and in compliance with the UK Animals (Scientific Procedures) Act, 1986.

### Tail skin samples

The MGP is a high-throughput, large-scale primary screen designed to detect changes involving large biological effect sizes. For most tests in the pipeline (including skin dysmorphology in 10-week-old mice and HF cycling and hair analysis in 43-day-old mice, presented in Fig. 2c), 7 male and 7 female mice are assessed per mutant line. However, for the epidermal wholemount screen (*n*=538 unselected knockout strains assessed for 557 alleles), two female mutants (range 1–9 mutant mice) were typically assessed per line. Female mice were chosen because they are less aggressive than males, thereby reducing the incidence of skin wounding and inflammation^54^ There is also sexual dimorphism in SG size and other skin-associated structures in mice^55^. Where possible, when abnormalities were detected in the primary screen, additional mice were screened to confirm the finding.

To establish a reference range for each strain assessed, epidermal wholemounts from different strains of WT mice fed on different diets (mouse breeder diet—chow; 5021, Labdiet) or high-fat diet—(21.4% crude fat content, Western RD, 829100, Special Diets Services) were analysed (*n*=168, *B6Brd;B6Dnk;B6N-Tyr*^*c-Brd*^ high fat; *n*=155, *C57BL/6N* high fat; *n*=56, *C57BL/6N* chow; *n*=18, *B6Dnk;B6N* chow; *n*=15, *129S/Sv*; *n*=12, *B6Brd;B6Dnk;B6N-Tyr*^*c-Brd*^ chow; *n*=11, *B6Dnk;B6N* high fat; *n*=10, *C3HeB/FeJ*; *n*=8, *CBACa;129P2; n*=8 *B6Jlco; n*=4, *B6Jlco;129P2; n*=4, *B6JIco;B6Brd;129P2-Tyr*^*c-Brd*^; *n*=4, *B6Brd;B6Dnk;B6N;129S5-Tyr*^*c-Brd*^; *n*=2, *129S5;B6N*; *n*=2, *AK BKS*). Wholemounts were analysed for each of the eight MP ontologies to establish the range of normal phenotypes.

### Epidermal wholemount staining

Intact tails were collected from ≥14-week-old mutant and WT mice of the same genetic background. Tails were labelled with unique IDs and either processed immediately or stored at 4 °C for less than 3 days. The skin was manually detached from the caudal vertebrae, and then divided into three parts for epidermal wholemount labelling, histology and re-genotyping.

The epidermal wholemount labelling procedure^10^ was modified to reduce the time required and allow large numbers of samples to be processed simultaneously. In brief, a scalpel was used to slit the tail lengthways. Pieces (0.5 × 0.5 cm^2^) of skin were incubated in 5 mM EDTA in PBS at 37 °C for 4 h. Forceps were used to gently peel the epidermis from the dermis as an intact sheet in a proximal to distal direction, corresponding to the orientation of the hairs, and then the epidermis was fixed in 4% paraformaldehyde (PFA; Sigma) for 1 h at room temperature. Fixed epidermal sheets were washed in PBS and stored in PBS containing 0.2% sodium azide at 4 °C for up to 1 year before labelling. Remarkably, epidermal sheets were still suitable for immunostaining after 3 years in storage.

Mouse monoclonal K14 (LL002) and K15 (LHK-15) antibodies^56^ were directly conjugated to Alexa Fluor 555 and 488 (A20174, A10235 Molecular Probes, Invitrogen), respectively, according to the manufacturer’s instructions. In brief, 50 μl 1 M sodium bicarbonate was added to 500 μl of 2 mg ml^−1^ purified protein. Alexa Fluor 555/488 reactive dye was added and incubated at room temperature for 1 h on a magnetic stirrer. Unconjugated dye was removed via column chromatography and conjugated antibody was eluted with 10 × elution buffer by gravity flow. Antibody dilutions (K14–1:200; K15—1:100) were optimized each time a new batch of directly conjugated antibodies was prepared. ab1653 (marker of scale epidermis) was used at 1:250 (Abcam, ab1653).

PFA-fixed epidermal sheets were placed in a 24-well tissue culture plate and blocked in PB (permeabilization & blocking) buffer (0.5% skimmed milk powder, 0.25% fish skin gelatin, 0.5% TritonX100, 1 × HEPES-buffered saline) for 30 min at 37 °C in a shaking incubator at 100 r.p.m. Directly conjugated K14 and K15 antibodies and DAPI diluted in PB were added to each well and incubated for 2 h in 37 °C in a shaking incubator at 100 r.p.m. Samples were washed three times with PBS/0.2% Tween solution at room temperature for 15 min per wash, and then gently rinsed with MilliQ water and mounted on glass slides in ProLong antifade medium (Invitrogen).

### Confocal microscopy

Image acquisition of wholemounts was performed using a Nikon A1 or Leica SP5 TCS confocal microscope. Signals in the DAPI, Alexa 488 and Alexa555 channels were merged. For the Nikon microscope, lasers of 405, 488 and 561 nm wavelength were used with the following objectives: 4X dry (Plan Fluor 4X/0.13 numerical aperture) with Z-range: ~425 mm; Z-step:~55; pixel dwell time (PDT): 5 mS and dry (Plan Fluor 10X/0.3 numerical aperture) with Z-range: ~125 mm; Z-step: 9.4; PDT=5 or 10.4 mS settings. 3D maximal projection images (1,024 × 1,024 d.p.i.) and 3D reconstruction movies were generated using NIS Elements version 4.00.04 (Nikon Instruments Inc.)

When imaging with the Leica SP5 confocal microscope, lasers of 405, 488 and 561 nm wavelength were used and Z-stacks were acquired at 100 Hz with an optimal stack distance and 1,024 × 1,024 d.p.i. resolution using 5X (0.07 dry), and (0.40 dry) HCX PL S-APO objectives. Leica Application Suite version 8.2.1 software (Leica Microsystems) was used for image acquisition and maximal projection.

### Skin phenotype calls

Phenotype calls were based on macroscopic observation of tails and confocal microscopy of epidermal wholemounts. In some mutant mice, pigmentation in the IFE was altered and this was recorded under bright-field illumination.

Any change in overall tail structure was called with an MP term ‘abnormal tail morphology (MP:0002111)’. Altered K14 expression (for example, reduced staining) was called as ‘abnormal epidermis stratum basale morphology (MP:0001231)’. Other changes in the IFE, including hyperproliferation (detected as increased basal layer density and envelopment of the HF infundibulum), and hypocellularity were also recorded under this term. Abnormal K15 expression and/or abnormal bulge morphology was called as ‘abnormal HF bulge morphology (MP:0009759)’. Any abnormalities in HF patterning (rows or triplets) were called with the term ‘distorted HF pattern (MP:0000384)’, while altered HF morphology, such as distorted follicles, was called as ‘abnormal HF morphology (MP:0000377)’. The hair growth cycle in tail epidermis is less highly synchronized than in the back skin and the mice analysed were not age matched^10^. Nevertheless, obvious disturbance of the hair growth cycle, such as all HF being in anagen or telogen, was recorded with the term ‘abnormal hair cycle (MP:0000427)’. Changes in SG size, number or morphology were recorded as ‘abnormal SG morphology (MP:0000647)’. Altered pigmentation was called with the term ‘abnormal epidermal pigmentation (MP:0009387)’.

Samples were examined and data were submitted without prior knowledge of genotype. When a mutant had no phenotype, the results were recorded on the tick sheet in the Sanger Mouse Genotype Portal (http://www.sanger.ac.uk/mouseportal). When a phenotype was abnormal, the information was summarized on the tick sheet and an illustrative 3D maximal projection image ( × 4, × 10 or both) was uploaded to the portal. Images were optimized globally for brightness, contrast and colour balance using Photoshop CS5 (Adobe image suite) before uploading. If there was incomplete allelic penetrance of a gene, this was recorded in the portal.

### Skin-specific *lacZ* reporter gene expression

*lacZ* reporter gene expression in skin was determined in wholemount tissue preparations from adult heterozygous mutant mice (6–12 weeks old)^57^. In brief, under terminal anaesthesia, mice were perfused with fresh cold 4% PFA, pH 8. Tissues were collected, fixed by immersion in 4% PFA for 30 min, and then incubated in *lacZ* staining solution containing 0.1% X-Gal (Invitrogen) at 4 °C for 48 h followed by post-fixation with 4% PFA at 4 °C. Tissues were cleared and stored in glycerol. Abdomen, pinna and tail skin were examined and staining was compared with a panel of standardized images. Images were taken using a Leica MZ16A microscope and processed by Imagic software. The pictures were annotated with MA terms before being released externally (http://www.sanger.ac.uk/mouseportal).

### Histochemistry

Tissue was fixed overnight in 10% neutral buffered formalin and was paraffin embedded. Sections were labelled with haematoxylin and eosin, antibody to Ki67 or Fontana–Masson stain by the histology core services of the Wellcome Trust-MRC Cambridge Stem Cell Institute or the CRUK Cambridge Research Institute. Sections were examined and photographed on an Axiophot microscope with an AxioCam HRc camera (Zeiss, Germany). Other antibodies were used at the following dilutions for immunofluorescence: mouse anti-FASN (SCBT; sc48357)—1:100; rabbit anti-Filaggrin (Covance; PRB-417P)—1:100; rabbit anti-Loricrin (Covance; PRB-145P)—1:100; and mouse anti-K10 (Covance; MMS-159S)—1:100. Alexa fluor (Life Technologies) dye-conjugated secondary antibodies were used at 1:250 dilution.

### Long- and short-range PCR and DNA sequencing

Tail DNA was prepared using a standard proteinase K and SDS lysis method^58^. Long- and short-range PCR were performed to re-confirm genotypes^12^. The following universal cassette and gene-specific primers were used for PCR amplification and DNA sequencing (see also Supplementary Fig. 4):

lacZ-genotype-For-5′-TGTATGAACGGTCTGGTCTTTG-3′;

lacZ-genotype-Rev-5′-CCAATCCACATCTGTGAAAGAA-3′;

L1-5′-CAACGGGTTCTTCTGTTAGTCC-3′;

LR-5′-ACTGATGGCGAGCTCAGACC-3′;

LRR-5′-GGTCTGAGCTCGCCATCAGT-3′;

R1-5′-AAGGCGCATAACGATACCAC-3′;

R2-5′-CCGCCTACTGCGACTATAGA-3′;

R2R-5′-TCTATAGTCGCAGTAGGCGG-3′;

R3-5′-GCGGATAACAATTTCACACAGGA-3′;

R4-5′-TGTAAAACGACGGCCAGT-3′;

PNF-5′-ATCCGGGGGTACCGCGTCGAG-3′;

Rad18-5′ HA-5′-TGTTGAATCCCTGCCTATCC-3′;

Rad18-3′HA-5′-CACCCTTGGCTTTTAAGTGG-3′;

Rad18-out5′ HA-5′-ACTTGGCTGGGAAGACATTG-3′;

Rad18-out3′ HA-5′-CCAGGATGTAACCCTGCACT-3′.

### GO and pathway network analysis

Enrichment of GO terms was performed using GoTermFinder ( http://www.go.princeton.edu/cgi-bin/GOTermFinder)^59^. The REVIGO web server ( http://www.revigo.irb.hr/) was used to generate GO scatter plots^60^. Localization of genes in the mouse karyotype was performed using Ensembl ( http://www.ensembl.org/Mus_musculus/Info/Index).

Direct (physical) and indirect (functional) protein associations were taken from the STRING database^31^ and members of each pathway were extracted from Reactome^61^. Interactions were assessed in both mouse and human. Enrichment analysis was performed using ToppGene^62^ on all 50 skin phenotype hits as well as the genes annotated to each specific MP term.

### Availability of biological resources and accessing the data

Data generated by this study can be viewed via the Sanger Mouse Resources Portal at http://www.sanger.ac.uk/mouseportal/ by entering the gene name of interest in the search menu. Users can then navigate through ‘Phenotyping Test based Heatmap’ to the ‘Tail Epidermis Wholemount’ section. Information, including ordering instructions, for all the mutant lines described in this study can be obtained either from the Sanger Mouse Resources Portal or the International Mouse Phenotying Consortium URL: http://www.mousephenotype.org/.

## Author contributions

F.M.W. and I.S. conceived the project. K.L performed experiments and had overall responsibility for the screen. E.H., T.D. and I.S. also performed experiments. V.V. contributed to the construction of data. D.P.S. performed computational network analysis. J.E., D.S., J.W., R.R. and K.P.S. managed the S.I.M.G.P for production and primary phenotyping of mutants. K.L and F.M.W. analysed the data and wrote the manuscript.

## Additional information

**How to cite this article:** Liakath-Ali, K. *et al.* Novel skin phenotypes revealed by a genome-wide mouse reverse genetic screen. *Nat. Commun.* 5:3540 doi: 10.1038/ncomms4540 (2014).

## Supplementary Material

Supplementary Figures and Table Supplementary Figures 1-7 and Supplementary Table 1

Supplementary Data 1 Genomic features of all 538 mutants analysed, showing mouse and human gene symbols, description, type of feature, Ensembl and Entrez IDs and chromosome localization.

Supplementary Data 2 Details of individual alleles (total 557) analysed, showing zygosity, sample number, allele type, coat colour, mouse colony ID, diet, age at death, core strain and genetic background.

Supplementary Data 3 Phenotype data of all individual mutants (total 538) analysed, including skin LacZ expression, Mouse skin phenotype annotated with MP terms, and human phenotype data mined from HPO database and OMIM and ORPHANET entries.

Supplementary Data 4 Detailed skin phenotype data with MP terms for all the 50 mutants identified in this study

Supplementary Movie 1 3D reconstruction generated from maximal projected wholemount confocal images of wild type tail epidermal wholemount. Green: K15 expression in the hair follicle bulge. Red: K14 expression. Blue: DAPI counterstain.
