# Supplementary material for: Novel skin phenotypes revealed by a genome-wide mouse reverse genetic screen
Source: Nat Commun. 2014 Apr 11;5:3540. doi: 10.1038/ncomms4540 (PMC3996542; doi:10.1038/ncomms4540)
Supplement: Supplementary Figures and Table — Supplementary Figures 1-7 and Supplementary Table 1 [file ncomms4540-s1.pdf]

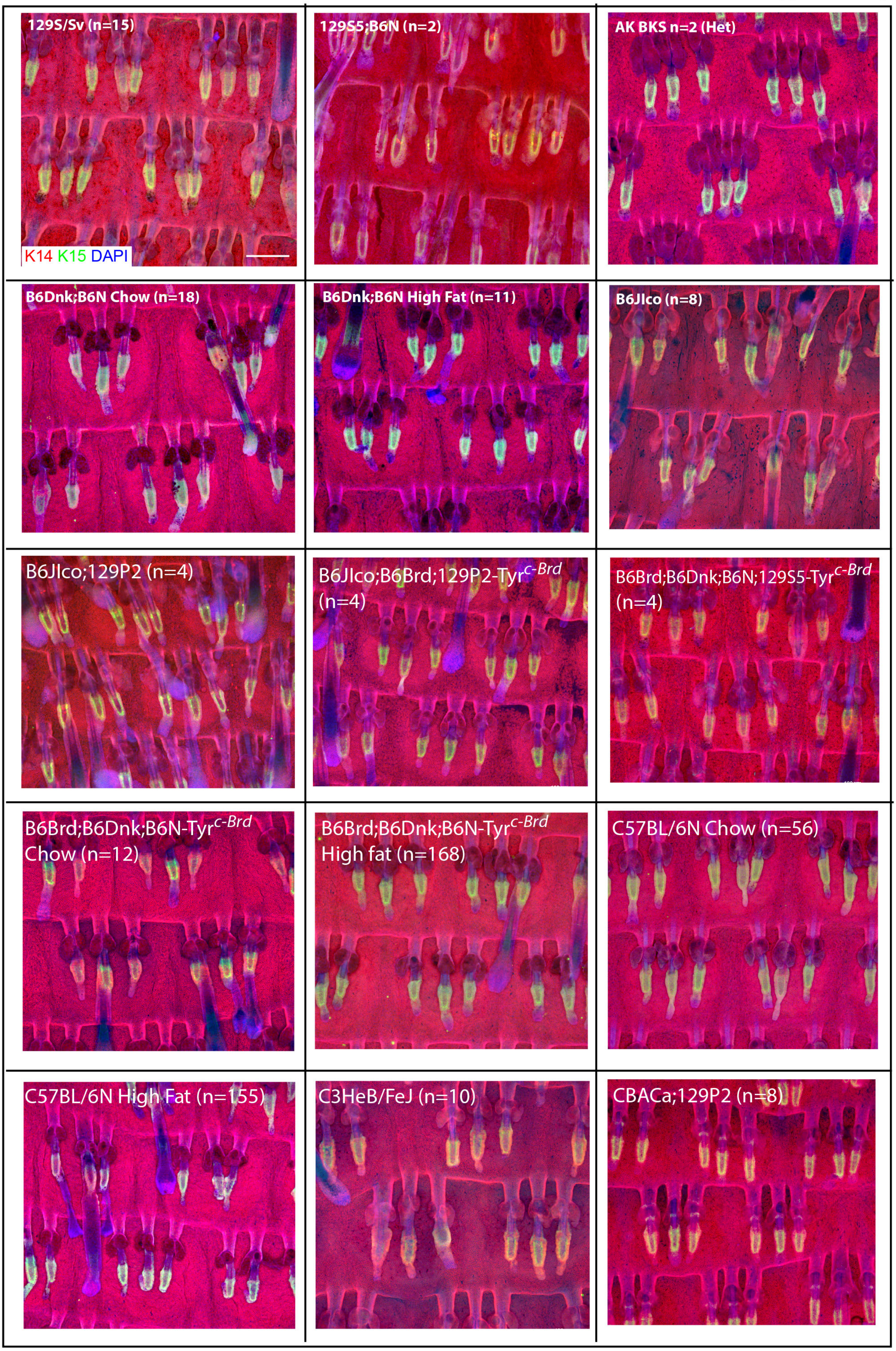

Supplementary Figure 1: Reference WT epidermal wholemounts
WT reference images showing panel of epidermal wholemounts from fifteen different core strains and number of samples analysed per strain. Scale bars 100µm.

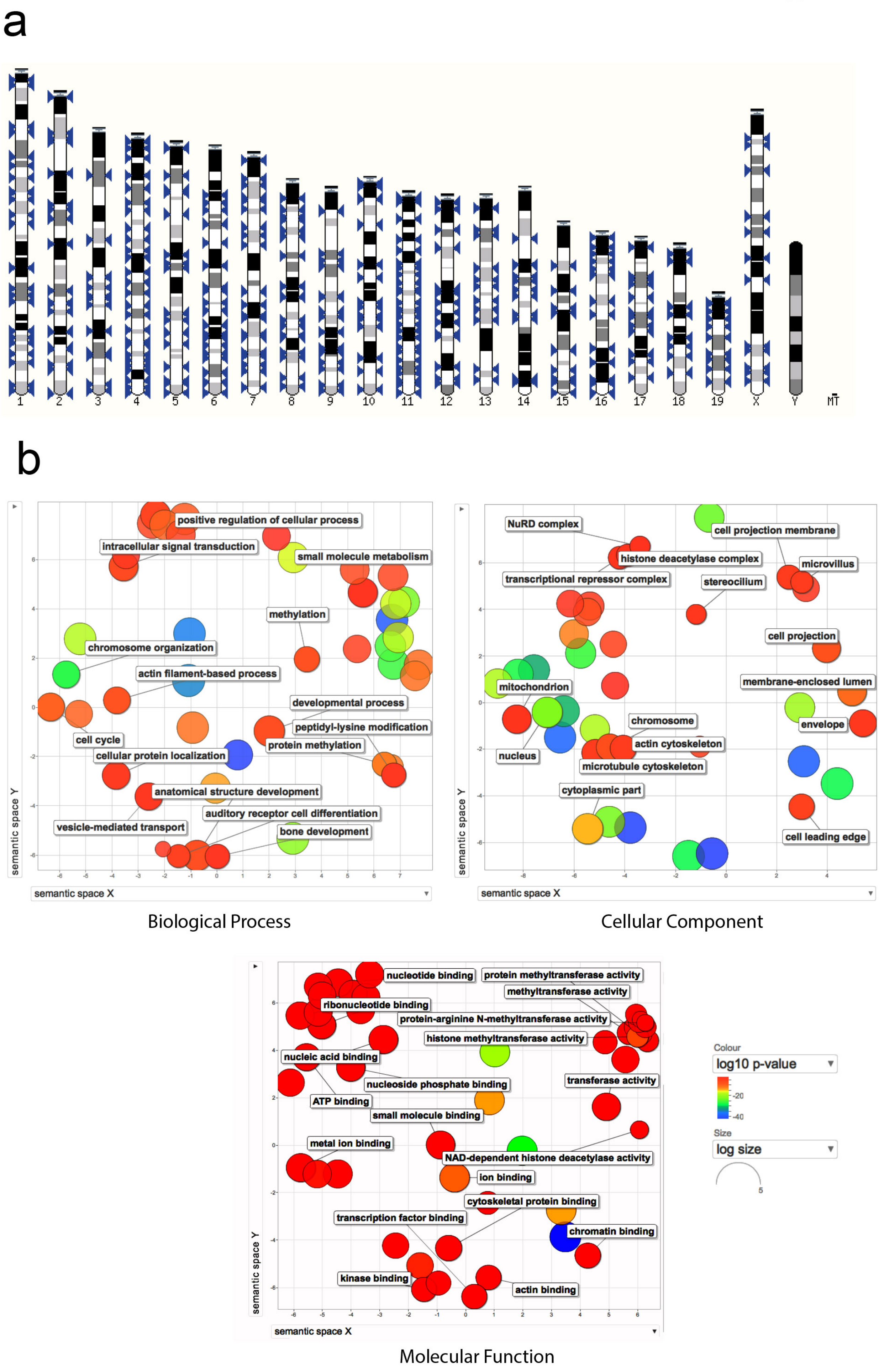

Supplementary Figure 2: Chromosome location and gene ontology of mutants

(a) Mouse karyotype showing all mutants analysed (blue arrowheads). (b) Bubble scatter plot representation of gene ontology terms of all mutants, generated using the REVIGO ontology tool to show enrichment of genes in different biological processes, cellular components and molecular function.

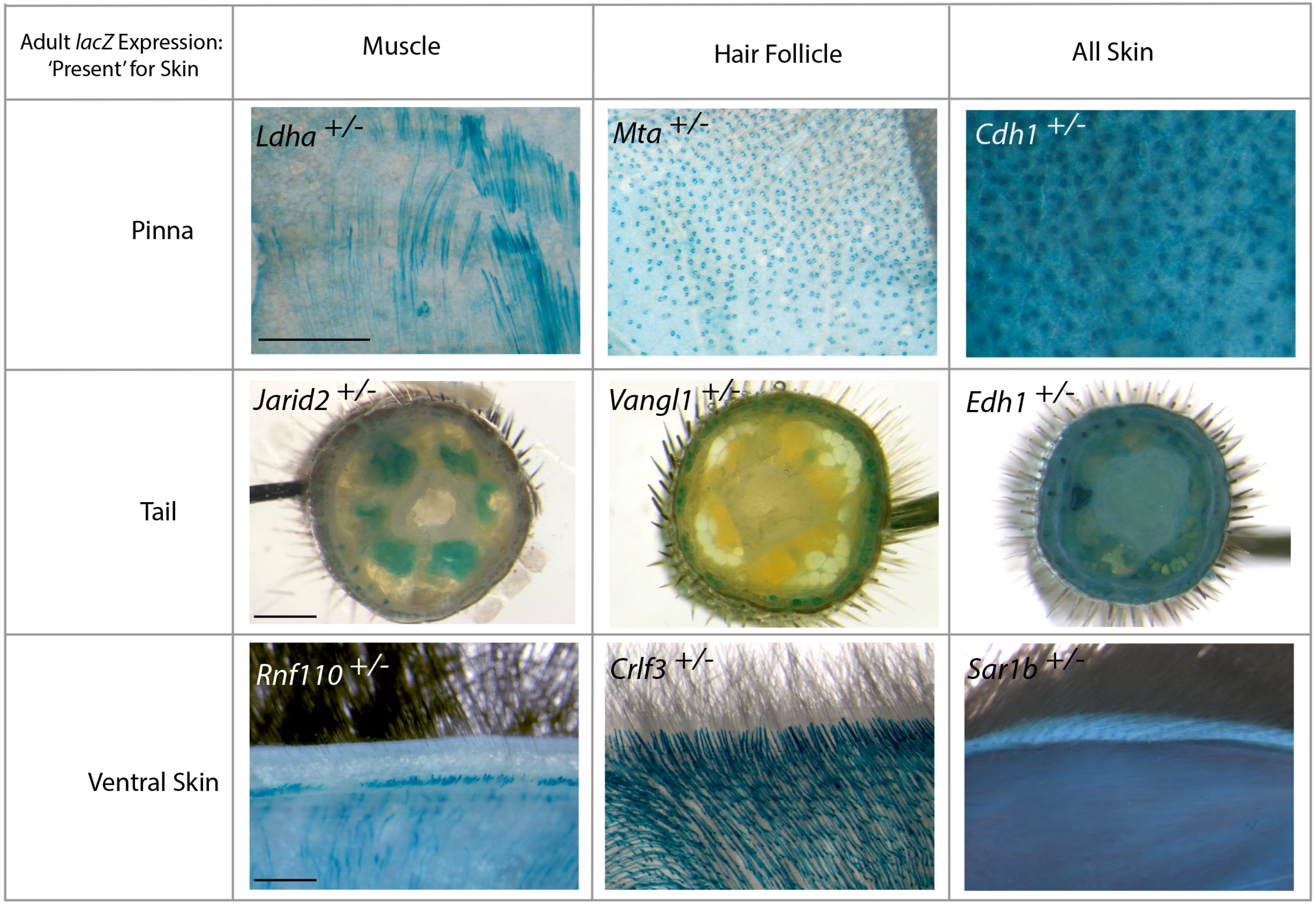

Supplementary Figure 3: Skin *lacZ* reporter expression calls
Representative images of LacZ reporter gene expression in adult skin from different body sites. LacZ expression was called as 'present' based on positive staining in muscle, hair follicles or whole skin. Scale bars 2.0mm (Pinna), 1.0mm (Tail and Ventral Skin)

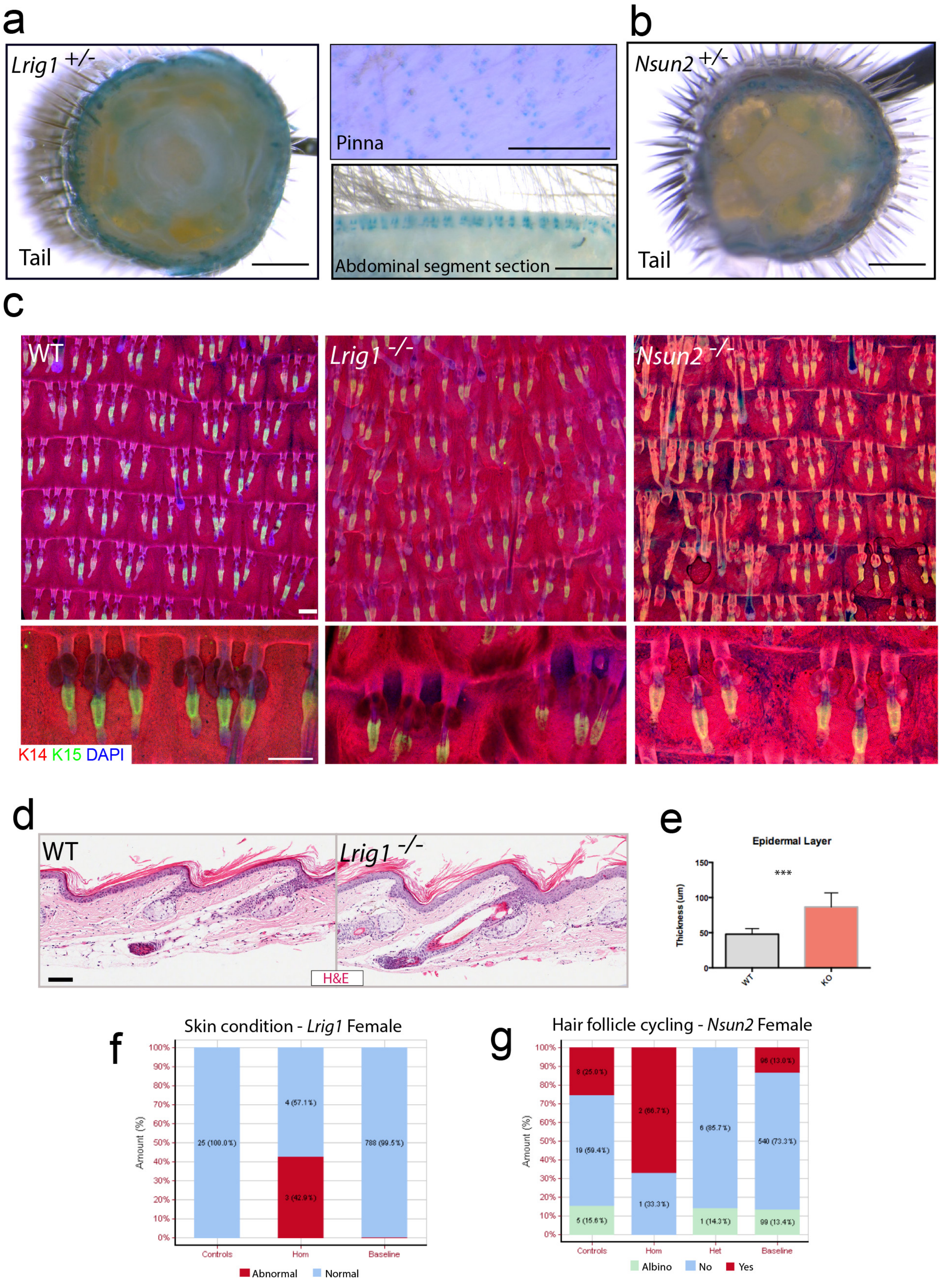

Supplementary Figure 4: *Lrig1* and *Nsun2* phenotypes
(a, b) LacZ reporter expression in *Lrig1*$^{-/-}$ (a) and *Nsun2*$^{-/-}$ (b) skin. (c) Epidermal wholemounts, showing abnormal stratum basale in both mutants and altered hair follicle pattern in *Lrig1*$^{-/-}$ mutant. (d, e) H&E staining (d) and quantification (e) showing increased thickness of *Lrig1*$^{-/-}$ mutant tail epidermis. Two samples with respective WT controls analysed. Thickness of each layer was quantified using Fiji (Image J) software. Stack bars showing results of unpaired t-test. Error bars denote mean with standard deviation; *** P 0.0002. (f, g) Bar charts showing incidence of scaly tail skin in *Lrig1*$^{-/-}$ mice (f) and hair cycle abnormalities in *Nsun2*$^{-/-}$, *Nsun2*$^{-/-}$ and control mice (g). See Fig. 4d legend. Test not performed on albino mice, but data show albino occurrence among baseline, control and heterozygote mice. Scale bars (a) 1.0mm (Tail and Abdominal segment section), 2.0mm (Pinna), (c, d) 100μm.

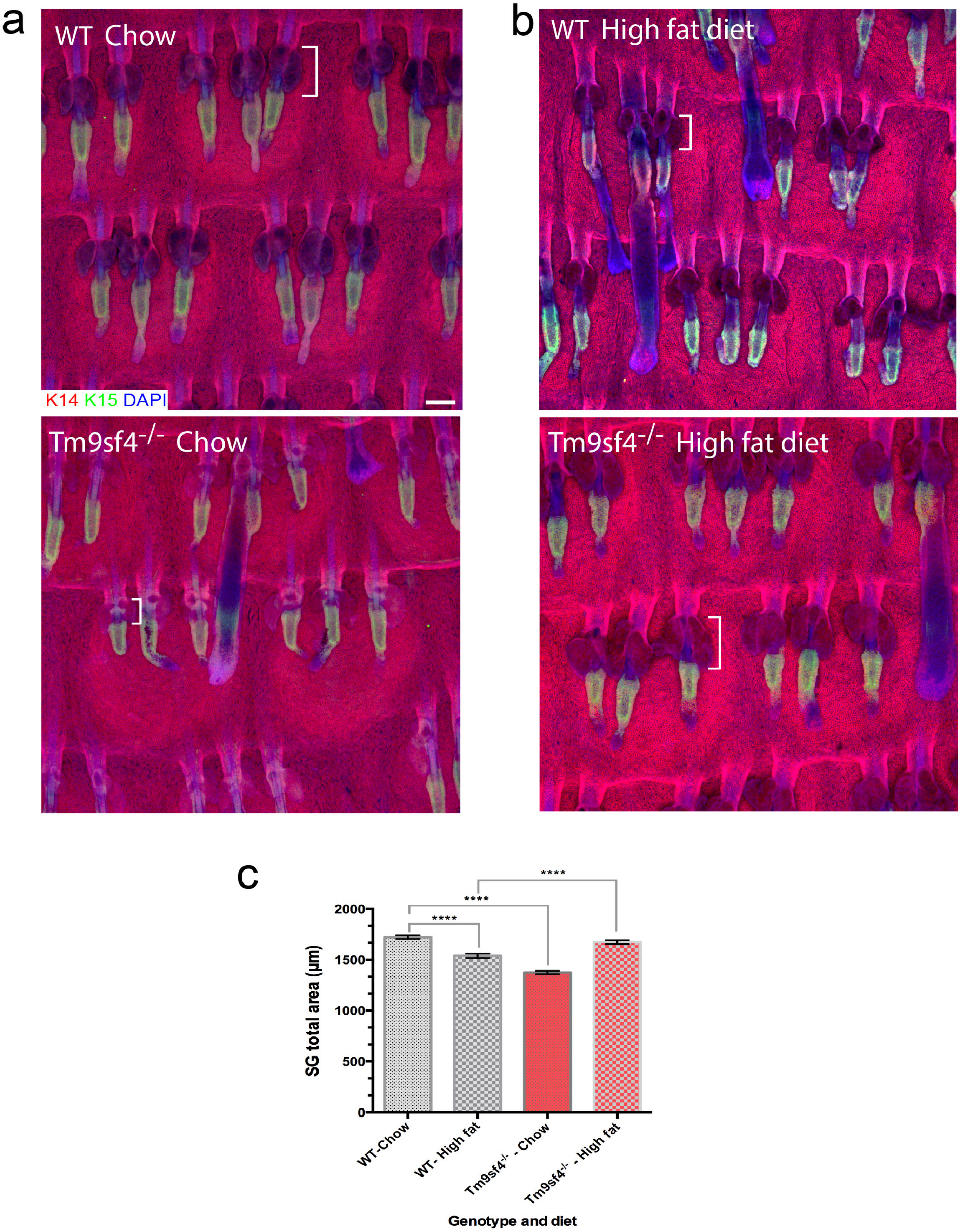

Supplementary Figure 5: Dietary influence on SG phenotype
(a, b) Epidermal wholemount images of *Tm9sf4*$^{-/-}$ mutant and WT mice fed on chow (a) or high fat diet (b). SGs are denoted by white brackets. (c) Quantification of SG size. Area of 50 telogen SGs were measured (n=2) using Fiji (Image J) software. Mann-Whitney test was performed to compare the significance of SG size difference between mutant and WT mice fed on chow and high fat diet. Error bars represent mean with standard deviation, **** P <0.0001, Error bars – s.e.m. Scale bars 100µm.

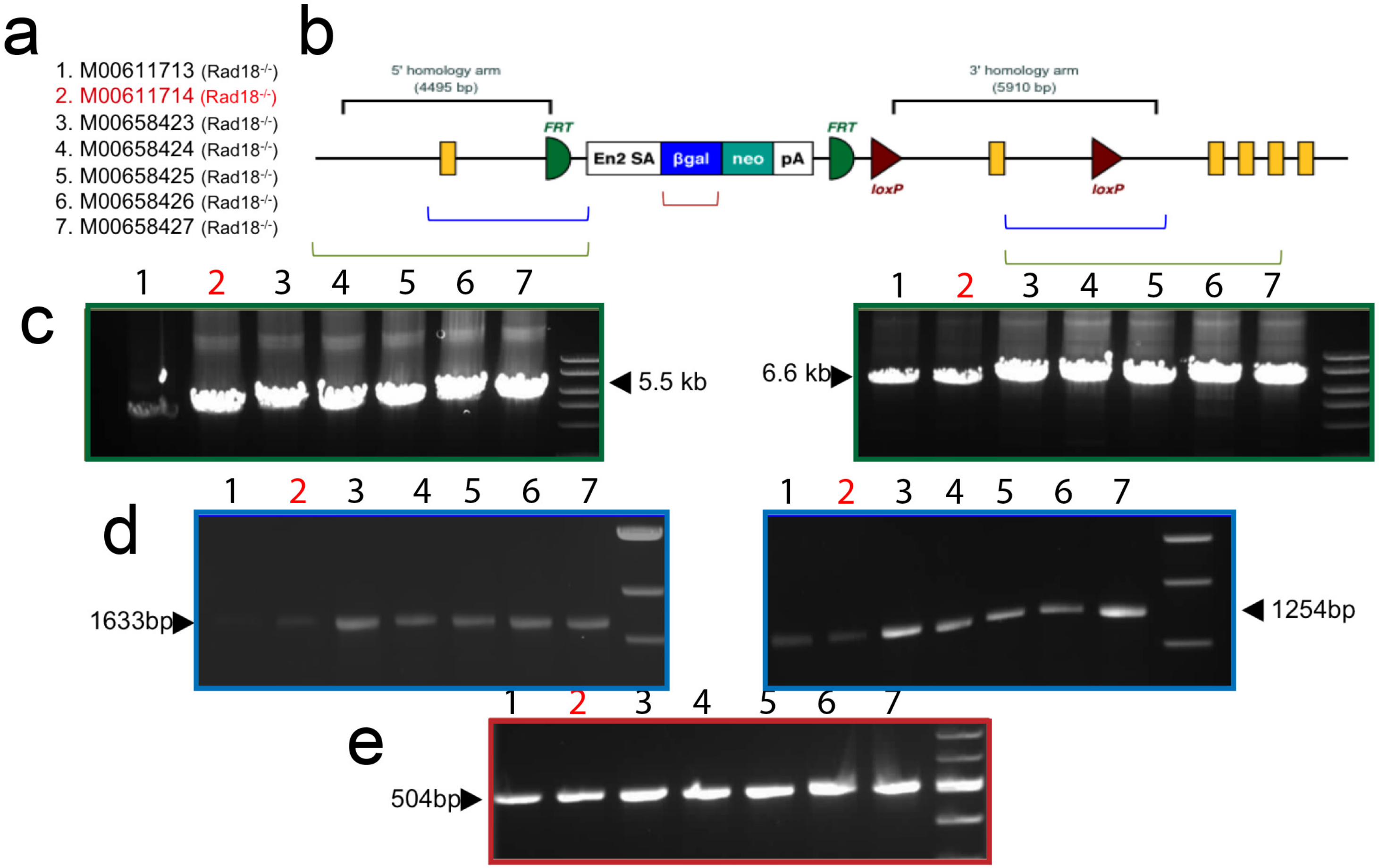

Supplementary Figure 6: Representative long range PCR re-genotyping (*Rad18*$^{-/-}$)
Re-genotyping Validation of genotype by long range PCR re-genotyping, using Rad18 as an example. (a) List of tail samples from *Rad18* null line. Sample with abnormal phenotype is indicated in Red. (b) KO structure of Rad18 allele. (c, d) Long range PCR with 5' and 3' gene specific and cassette specific primers. (e, f) Short range PCR with 5' and 3' gene specific and cassette specific primers. (g) Short range PCR with LacZ primers.
Gel borders (c-e) are colour coded to match the corresponding regions of the construct in (b).

Supplementary Figure 7

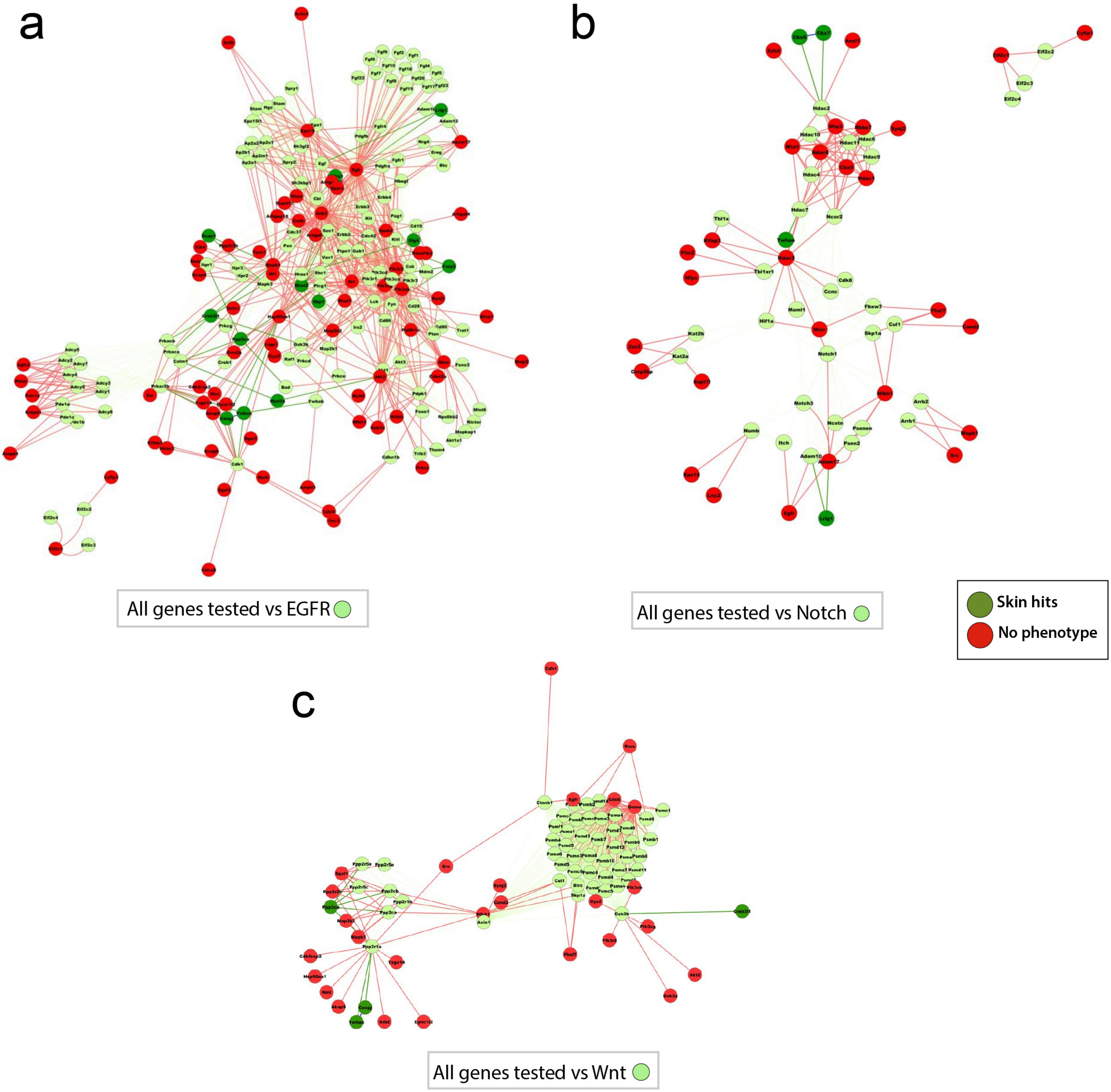

Supplementary Figure 7: Pathway interactions of all mutants

Interaction of all 538 mutant genes (red: no skin phenotype; green: skin phenotype) from the screen with Egfr (a), Notch (b) and Wnt (c) pathway genes in mouse.

Supplementary Table 1: Enrichment analysis on 50 phenotypic hits

Enriched ID	Enriched term	Bonferroni FDR adjusted p-value	Matches in query / Matches in genome	Matched genes
GO:0051904	pigment granule transport	6.104E-3	2/17	MYO7A, MYO5A
GO:0033059	cellular pigmentation	2.662E-2	2/35	MYO7A, MYO5A
MP:0001324	abnormal eye pigmentation	2.460E-2	3/120	MYO7A, MYO5A, ABCA4
MP:0009389	abnormal extracutaneous pigmentation	2.649E-2	3/123	MYO7A, MYO5A, ABCA4
PMID:14978221	The role of Rab27a in the regulation of melanosome distribution within retinal pigment epithelial cells.	1.439E-4	2/3	MYO7A, MYO5A
MP:0004324	vestibular hair cell degeneration	4.214E-2	2/10	USH1C, MYO6
